# The Importance of Telomere Shortening for Atherosclerosis and Mortality

**DOI:** 10.3390/jcdd7030029

**Published:** 2020-08-06

**Authors:** Wolfgang Herrmann, Markus Herrmann

**Affiliations:** 1 Department of Clinical Chemistry, Medical School of the Saarland University, 66421 Homburg, Saar, Germany; w-herrmann@gmx.de; 2 Clinical Institute of Medical and Chemical Laboratory Diagnostics, Medical University of Graz, 8036 Graz, Austria

**Keywords:** telomere, atherosclerosis, carsiovascular disease, age, inflammation, B vitamins

## Abstract

Telomeres are the protective end caps of chromosomes and shorten with every cell division. Short telomeres are associated with older age and adverse lifestyle factors. Leucocyte telomere length (LTL) has been proposed as a biomarker of biological age. The shortening of LTL with age is the result of the end-replication problem, environmental, and lifestyle-related factors. Epidemiologic studies have shown that LTL predicts cardiovascular disease, all-cause mortality, and death from vascular causes. Age appears to be an important co-variate that explains a substantial fraction of this effect. Although it has been proposed that short telomeres promote atherosclerosis and impair the repair of vascular lesions, existing results are inconsistent. Oxidative stress and chronic inflammation can both accelerate telomere shortening. Multiple factors, including homocysteine (HCY), vitamin B6, and vitamin B12 modulate oxidative stress and inflammation through direct and indirect mechanisms. This review provides a compact overview of telomere physiology and the utility of LTL measurements in atherosclerosis and cardiovascular disease. In addition, it summarizes existing knowledge regarding the impact of oxidative stress, inflammation, HCY, and B-vitamins on telomere function.

## 1. Introduction

The progressive deterioration of genomic integrity and genomic instability are key aspects of aging [1,2]. Telomeres represent the protective end caps of chromosomes and are of critical importance for the preservation of our genome. Their length has been considered a ‘biological clock’ that is useful for the estimation of an individual’s biological age [3,4] and to estimate the risk for age-related diseases and mortality. Telomeres are evolutionary conserved and were first described by Hermann Müller [5]. Together with Barbara McClintock, he identified their protective function [5,6]. They are composed of a non-coding, repetitive DNA sequence that is linked to proteins that form the shelterin complex [3,4,7,8]. For the most part of its length, telomeric DNA is double-stranded and is expanded by a short, G-rich single-stranded overhang at the 3′-OH end. Supported by the shelterin proteins, telomeric DNA folds into a three-dimensional structure that is important for telomere function and protection [3,4,7,8]. It is well established that telomeres continuously shorten with increasing age [3], which weakens their protective properties and limits the proliferative potential of mitotic cells.

Telomeres occur in all nucleated cells. At birth, every person has a specific telomere length (TL) that ranges between 5 to 15 kb [3,9]. Throughout life, telomeres shorten continuously with a rate between 20–50 bp due to factors, such as the end-replication phenomenon, oxidative stress, and others. The end replication problem refers to the inability of DNA polymerase to fully replicate the 3′ end of chromosomes, which leads to the loss of a small telomeric DNA fragment with every cell division [10]. When telomeres become critically short, they lose their protective properties and send cells into senescence, or cause cell death [10]. Telomere shortening rates, and consequently, average TL vary amongst different tissue types, which is at least partly explained by tissue-specific proliferation rates [9]. In dividing cells, the end replication problem is the principal driver of telomere shortening that can be modified by other factors, such as oxidative stress or inflammation [4,11]. In post-mitotic cells, instead, oxidative stress can directly damage telomeric DNA and drive cells into senescence. Despite tissue-specific proliferation rates, TL in different tissues and peripheral blood leucocytes are correlated [9,12,13]. Therefore, leucocyte telomere length (LTL) has been proposed as a surrogate marker of TL in the entire body [9,12,13]. Considering that most studies in the area of vascular disease analyzed telomeres in peripheral blood leucocytes, this review focuses primarily on LTL and mentions TL in other cell types only when necessary.

The initial results from observational studies, such as the population-based Bruneck study, suggested a markedly higher all-cause mortality in individuals with short LTL [14]. Considering that cardiovascular disease is still the main cause of death in developed countries, these results have triggered substantial interest in the role of telomeres in atherosclerosis and cardiovascular disease. Disappointingly, the first studies that focused on cardiovascular mortality found no [11,15] or only small effects in distinct sub-groups [16,17]. However, over the last decade, our knowledge about the role of telomere dysfunction in atherosclerosis and cardiovascular disease has increased substantially. This review article aims to provide a summary of the existing literature until May 2020.

## 2. Telomere Length and Age

**Figure 1:**
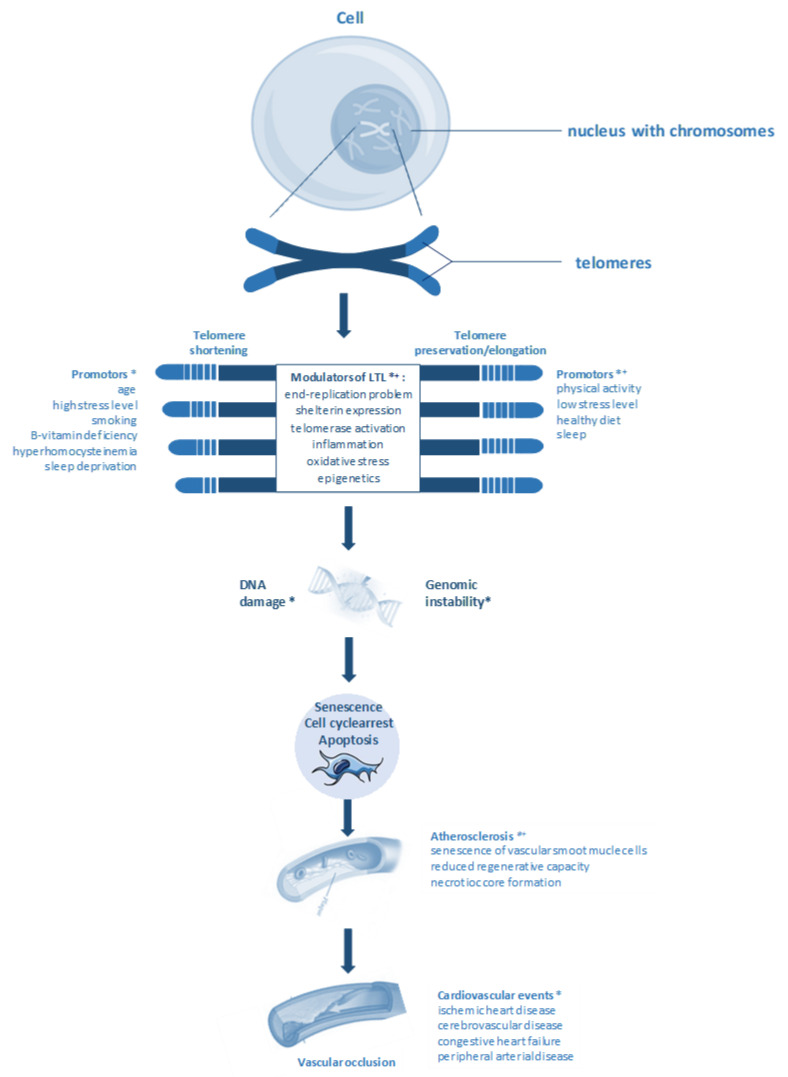
Telomere shortening causes genomic instability, which (1) triggers cell-cycle arrest, senescence, and apoptosis of vascular smooth muscle cells, (2) reduces the regenerative capacity of blood vessel tissue, and (3) promotes the formation of a necrotic core in atherosclerotic plaques. Ultimately, this increases the risk of cardiovascular events. Telomere length could be preserved through lifestyle interventions, such as regular physical activity, low-stress levels, healthy diet, and sleeping patterns. * supported by correlation studies, ^+^ supported by functional studies in vivo or in vitro.

Age and numerous lifestyle factors including physical activity, body mass index, alcohol consumption, hormone replacement therapy, dietary intake of antioxidants, trace elements, and vitamins, chronic inflammation, socioeconomic status, stress, and paternal age, are important determinants of LTL, Figure 1 [18,19]. The mean LTL at birth is approximately 11 kbp and declines to less than 4 kbp in elderly individuals. However, telomere shortening appears to vary substantially between individuals and different studies. A meta-analysis by Müezzinler et al. [20] summarized 124 cross-sectional and five longitudinal studies, where subjects were analyzed repeatedly over an extended period of time. The cross-sectional studies covered an age range from 0 to 104 years, and the number of participants ranged from 23 to 12,409. The few longitudinal studies combined included a total of 1365 subjects with an age range from 19.9 to 91 years and a mean follow-up of 8.7 years. Müezzinler et al. showed that young adults have an LTL between 7 and 9 kbp, whereas in individuals between 40 and 60 years, the LTL varies from 6 to 8 kbp [20]. Above 60 years, most studies found an even lower LTL that ranged between 4.5 and 6.5 kbp. However, a few studies reported considerably longer telomeres in leucocytes with 8.5 to 10 kbp. Own results from >3300 participants of the LUdwigshafen RIsk and Cardiovascular Health (LURIC) study also support a lower LTL with increasing age (r = −0.09; *p* < 0.001) [21]. In line with the results from LURIC, in their meta-analysis, Müezzinler et al. showed a significant inverse correlation between the mean age of each study cohort and the respective mean LTL across all cross-sectional studies for both absolute (r = −0.338, *p* < 0.0001) and relative (r = −0.295, *p* = 0.0088) LTL. From these results, a yearly telomere loss of 24.7 base pairs (bp)/year and 0.010 T/S ratio/year (telomere/single-copy gene) (*p* = 0.0071) was estimated. The weighted means of the within-study correlation between age and LTL were in a similar order of magnitude (−0.380 and 21.91 bp/year). For studies with more than 1000 participants, the loss rate was 20–30 bp/year. No statistically significant differences were observed between men and women.

The few longitudinal studies confirmed a decrease of LTL with age [22,23,24,25]. The yearly telomere loss rate, evaluated in only three studies, ranged from 32.2 to 45.5 bp/year. No sex differences were observed. However, ethnic differences appear to exist. African Americans have longer LTL compared to White Americans [22]. LTL tracking in 67 children and 99 of their parents over a period of approximately 14 years showed that telomere attrition in adults is only half of that in children (20.3 vs. 40.7 bp/year) [26].

Although epidemiologic studies show a decrease of LTL with age, in some individuals, telomeres appear to elongate over time. In the longitudinal Bogalusa Heart Study, 16% of all participants showed telomere elongation over a period of seven years, but when a longer period of 12 years was considered, this figure decreased to 1.5% [22]. This implies that over the time span of a few years, LTL could increase or decrease depending on the individual presence of LTL-modifying factors [27,28,29]. However, over longer periods, these effects are overcome by the strong influence of age. In 4576 subjects of the Copenhagen City Heart Study with a 10 year follow-up, 56% showed a loss of LTL, whereas 44% gained telomere length. In this cohort, the ten-year maximum loss and gain were −8.406 and +7.278 bp, respectively [30]. Participants that gained or lost telomere length over the 10 years follow-up period showed similar baseline characteristics. However, baseline LTL was inversely associated with the change in LTL and explained 29% of the variation over 10 years [30]. Although telomere elongation is a common finding in longitudinal studies [22,27,30,31], the discussion of whether this is a real biological phenomenon or an analytical artifact is still ongoing and large longitudinal studies with sufficiently long follow-up in different age groups are required in order to answer this important question. Furthermore, Martinez-Delgado et al. [28] reported that patients with hereditary breast cancer presented shorter telomeres than sporadic breast cancer patients and the control population, suggesting a modifier effect of the Breast Cancer (BRCA) 1 and BRCA2 genes on telomere-length. Additionally, it has also been shown that chemotherapy exerts a transient telomere shortening effect (around two years) that varies depending on the drug combination [29]. In familial cases, LTL shortening was only seen in treated, but not untreated patients. However, within two years, the LTL of treated patients returned to pre-treatment levels.

## 3. Telomere Length, Cardiovascular Disease (CVD), and Mortality

On the basis of the association between LTL and age, it has been speculated that LTL might also be linked to mortality [11,32,33]. Therefore, several longitudinal studies have investigated if LTL predicts mortality [21,33] and age-related diseases, such as CVD [10,14,34,35], acute myocardial infarction (AMI) [10,14], atherosclerosis [10], hypertension [10], and type 2 diabetes mellitus [10]. We and others have shown that LTL is a strong predictor of mortality [14,15,17,36,37,38]. For example, in the population-based Bruneck study, subjects with the longest telomeres had the lowest risk of dying during 10 years of follow-up [14]. In the LURIC cohort, we found a lower relative telomere length (RTL) in the leucocytes of the 995 patients who died during the 10 years follow-up period, compared to 2321 alive (2.0405 vs. 2.2050; *p* = 0.015) [21]. Furthermore, survival was lowest amongst participants in the 1st quartile of RTL. The Cox regression analysis for all-cause mortality showed that a lower RTL is associated with increased all-cause mortality, even after adjustment for major cardiovascular risk factors, such as sex, LDL cholesterol, HDL cholesterol, triglycerides, body mass index, lipid-lowering therapy, blood pressure, diabetes mellitus, smoking, coronary artery disease (CAD), high-sensitive C-reactive protein, and estimated glomerular filtration rate. Similar to our results, in other large-scale studies, subjects with the shortest telomeres had a 17–66% increase in mortality risk when compared to subjects with the longest telomeres [14,15,17,36,38].

Further analyses revealed that short telomeres are also associated with cardiovascular risk factors, including CVD, AMI, atherosclerosis, hypertension, and diabetes mellitus [14,34]. In the prospective West of Scotland Coronary Prevention Study (WOSCOPS), including 1542 men with no history of AMI, individuals with the shortest telomeres (1st quartile) had a 44% higher incidence of CVD compared to individuals in the 4th quartile [34]. In a small prospective cohort study of 290 patients surviving recent AMI, LTL measured on admission was a strong predictor of all-cause [hazard ratio (HR) [95% confidence interval (CI)]: 3.21 [1.46–7.06], *p* = 0.004] and cardiovascular mortality (HR [95% CI]: 3.96 [1.65–9.53], *p* = 0.002) 1 year after AMI [37]. In the Bruneck study, subjects with the longest telomeres had the lowest risk of developing CVD, stroke, MI, and vascular death during a follow-up period of 10 years [14]. Not surprisingly, in the LURIC study, subjects with the shortest telomeres had the highest risk of dying from CVD [21].

While the majority of studies showed a significant association between LTL, mortality, and CVD, some did not [16,32,33]. In a Swedish study with 2744 elderly men, LTL was neither related to all-cause mortality nor to CVD mortality [33]. Similarly, Bischoff et al. did not find an association between LTL and survival among 812 elderly subjects from three different Danish cohorts [32]. It should be mentioned that many studies have investigated low-risk populations with a limited number of events, whereas others, such as LURIC, focused on medium to high-risk cardiovascular patients. This may at least partially explain inconsistent results between individual studies. For example, in the Bruneck study, only 88 out of 800 subjects experienced a CVD event during follow-up, which is much less than in LURIC [14].

Therapeutic strategies based on telomerase activation are being under investigation in order to treat telomere-associated diseases, like age-related diseases and telomeropathies [39]. It has been reported that sex hormones activate TERT transcription [40] and that testosterone therapy in mice suffered from aplastic anemia with short telomeres was able to upregulate telomerase expression and to restore TL, and to prolong their lifespan [41]. In humans, the administration of the synthetic androgen Danazol to patients with telomeropathies resulted in LTL elongation and improvement of hematologic parameters [42]. Moreover, therapeutic interventions based on telomerase-based gene therapy are currently being tested in mouse models to improve health and extend their lifespan [43]. Telomerase activation is seen as an encouraging strategy to prevent or treat age-related diseases [43,44,45,46]. Thus, experimental data support the feasibility of telomerase activation strategies to prevent the accumulation of critically short telomeres and resulting consequences [39]. However, there is an ongoing debate as to whether therapeutic telomerase activation increases cancer risk or not [47,48,49]. At present, experimental evidence exclusively comes from experiments in mice. Human studies are still lacking. Therefore, more data is needed to estimate the potential risks associated with telomerase activating therapies.

## 4. Telomere Length and Atherosclerosis

Atherosclerosis, a condition characterized by arterial plaque formation with subsequent narrowing of arteries and increased stiffness of the vessel wall, is the primary cause of CAD and stroke [50]. Typically, the disease progresses for many years unnoticed, and patients are unaware that they are moving towards a serious health condition with a high risk of morbidity and mortality. Considering the increased CVD risk in individuals with short LTL, it can be expected that LTL is also related to markers of subclinical atherosclerosis, such as intima-media thickness and arterial elasticity. Several studies have investigated LTL and subclinical atherosclerosis in different cohorts [51,52,53,54]. The results of cross-sectional studies are inconsistent [51]. For example, in 1062 individuals (496 men, 566 women, 33–86 years) of the Framingham study, age-adjusted LTL was a strong predictor of the internal carotid artery intima-media thickness (ICA-IMT) in obese men (body mass index > 30 kg/m^2^). However, in the entire cohort, the relationship was rather weak, and no significant association was observed with common ICA-IMT or with carotid artery stenosis [51]. Another study with subjects aged >40 years reported an inverse association of LTL with common carotid IMT [55]. However, in hypertensive or elderly men [54,56], shorter LTL was correlated with carotid artery plaque formation. In addition, LTL was negatively associated with coronary artery calcification in a low-risk cohort free of CVD [57]. Fernández-Alvira et al. conducted another cross-sectional study with 1459 volunteers without established cardiovascular disease (58% men, 40–54 years) [58]. After adjustment for cardiovascular risk factors, short LTL was not a significant predictor of total and femoral plaques [58]. Likewise, in the Asklepios study, LTL was neither a significant determinant of intima-media-thickness nor plaque presence [52]. In the very few longitudinal studies conducted so far, LTL shortening was associated with all-cause mortality in patients with stable coronary artery disease [59] or type 1 diabetes mellitus [60]. The prospective Strong Heart Study examined whether LTL predicts incident carotid atherosclerosis and progression in a cohort of 2819 American Indians who were free of overt cardiovascular disease at baseline [50]. During an average follow-up period of 5.5 years, individuals with the shortest LTL had a 49% and 61% higher risk for incident plaques and plaque progression than those with the longest LTL. In the prospective Bruneck study, short LTL was a predictor of advanced, but not early, atherogenesis [14]. Table 1 provides an overview of essential studies that investigated the role of telomeres in atherosclerosis and cardiovascular disease.

## 5. Telomere Shortening and CVD in Pre-Clinical Models

Commonly used animal models, such as mice, do also exhibit age-related telomere shortening in blood leucocytes and solid organ tissues (e.g., myocardium, liver, aorta) [68,69,70]. However, compared to humans, telomere shortening in mice is rather slow. In C57/Bl6 mice, Werner et al. detected a significant reduction in TL of blood leucocytes and cardiomyocytes after 18 months, but not earlier [68,69]. The age-related shortening of telomeres goes along with reduced expression of shelterin proteins, such as Telomeric Repeat Factor (TRF) 1 and TRF2 [70], increased apoptosis, up-regulation of senescence-related proteins (Chk2, p53, p16), and cell-cycle arrest [68,69,71]. In contrast, the preservation of TL through overexpression of TERT delays the process of aging and extends the life span [72,73]. Moreover, telomerase reactivation reverses tissue degeneration in telomerase deficient mice [74].

The importance of intact telomeres for cardiovascular health is supported by exercise studies. Cardiomyocytes of exercising mice are characterized by the preservation of TL, an up-regulated myocardial expression of telomerase and shelterin proteins, and a reduction in apoptosis and cell-cycle arrest [68,69,71,75]. Furthermore, physical activity down-regulates the senescence-related proteins Chk2, p53, and p16. Werner et al. also demonstrated that regular running exercise ameliorates the cardiotoxic effects of doxorubicin [69]. In vivo studies in mice suggest that the beneficial cardiac effects of regular exercise are primarily mediated by Telomerase Reverse Transcriptase (TERT), Epithelial NO Synthase (eNOS), and Insulin-like Growth Factor 1 (IGF-1). Interestingly, already a single bout of exercise has been shown to increase TRF1 and TRF2 protein levels and the expression of DNA-repair and -response genes (Chk2 and Ku80) [75]. The exercise-induced increase in shelterin gene expression is believed to depend on the duration, intensity, and type of exercise [75]. However, the rapid increase of shelterin expression in response to a single exercise session does not necessarily lead to a prompt increase in telomerase activity (TA). A persistent myocardial telomerase activation, which appears to be essential for the cardioprotective effects of physical activity, may take several weeks of regular training [68,69].

While exercise preserves TL in mice, hypercholesterolemia has been found to induce oxidative stress, shorter telomeres, an abnormal cell cycle status, and an impaired reconstitution capacity of hematopoietic stem cells, indicating accelerated aging in these cells [76]. Wang et al. demonstrated that impaired TRF2 function in vascular smooth muscle cells (VSMC) of genetically modified mice increases atherosclerosis and necrotic core formation, whereas overexpression of functional TRF2 protects against atherosclerosis independent of secretion of senescence-associated cytokines [77]. Based on these observations, it has been speculated that VSMC senescence promotes atherosclerosis and plaque vulnerability.

## 6. B-vitamins, Homocysteine, Telomere Length, and CVD

Prospective studies from around the world have revealed elevated plasma homocysteine (HCY) as a risk factor for mortality and atherosclerosis [78]. AMI occurs as a result of atherosclerotic plaque rupture and the emergence of a thrombus. Amongst others, the non-protein forming amino acid HCY has been identified as an independent risk factor for cardiovascular diseases [79,80]. A rise of 5 μmol/L in plasma HCY level increases the risk of coronary artery disease (CAD) from 20% to 50% [61]. Whatever the cause is, mild and moderate hyperhomocysteinemia (HHCY) (13 to 25 μmol/L) is strongly associated with stroke, obstructive CVD [81], restenosis, heart failure and major adverse cardiac events (death, reinfarction, restenosis) after the percutaneous coronary intervention (PCI) [82], and with all causes of death [83]. The effects of HCY in CVD appear to be a consequence of increased oxidative stress in the vascular endothelium resulting in reduced NO availability, impaired vasodilatation, inflammatory reactions with endothelial injury, and dysfunction [84,85]. For example, Chambers et al. demonstrated a rapid onset of endothelial dysfunction in response to acute HHCY, which was reversible through the administration of the antioxidant vitamin C [86]. These effects are at least partially caused by reduced availability of the vasodilator NO. HHCY and oxidative stress reduce the availability of NO through NO inactivation and uncoupling of endothelial nitric oxide synthase (eNOS) [87,88,89]. The oxidative reduction of NO is accompanied by a decreased expression of cellular glutathione peroxidase (GPx-1), and increased production of reactive oxygen species (ROS) [89]. In addition to the reduced NO availability, increased ROS production in HHCY patients promotes endothelial injury and dysfunction through pro-inflammatory pathways, the proliferation of vascular smooth muscle cells, and the production of extracellular matrix [90]. Another HCY-induced oxidative damage mechanism is the upregulation of human p66shc, a protein that promotes oxidative stress and endothelial dysfunction via hypomethylation of specific CpG dinucleotides in the p66shc promoter region [91]. In cell culture experiments with cardiomyocytes, Hcy-supplemented medium reduced contractility and promoted apoptosis. These effects were accompanied by an activation of p38 mitogen-activated protein kinase (MAPK), a decreased expression of thioredoxin protein, and an increase of ROS [92]. In human umbilical vein endothelial cells (HUVECs), HCY increased malondialdehyde (MDA) synthesis and the expression of intercellular adhesion molecule 1 (ICAM-1) [93]. In contrast, the expression of superoxide dismutase 2 (SOD2) and eNOS was inhibited by HCY. The role of HCY as a promotor of oxidative imbalances is further supported by the observation that supplementation of HUVECs with folic acid and vitamin B12 attenuated the HCY-induced effects on MDA, SOD2, eNOS, and ICAM-1. Furthermore, DNA total methylation level in HCY treated cells was significantly decreased, while DNA total methylation levels increased after treatment with folic acid and vitamin B12. Similar effects were observed for the methylation level of sorbin and SH3 domain-containing protein 1 (SORBS1), and discussed that HCY induces oxidative stress via SORBS1 methylation.

Due to its role in one-carbon metabolism, HCY is considered a functional marker of folate, vitamin B6, and B12 availability. Deficiencies of one or more of these vitamins can hamper the detoxification of HCY and result in hyperhomocysteinemia (HHCY), which leads to oxidative imbalance and an excess ROS, such as peroxides and free radicals. ROS can assault the DNA leading to base damage, DNA strand breaks, and accelerated telomere shortening [78]. Existing studies that explored the relationship between HCY and telomere shortening are mainly of cross-sectional nature and yielded inconsistent results. While some studies reported an inverse association between telomere length and HCY [62,94,95], others did not [63,96,97]. In the prospective LURIC study, plasma HCY was identified as an independent predictor of all-cause mortality [66]. The unadjusted HR for death from all causes and from CVD was nearly three times higher in the fourth quartile of HCY than in the first quartile. In addition, vitamin B6 was also a strong predictor of death. In the crude model, subjects with the highest vitamin B6 concentrations (4th quartile) had a 59% lower risk to die during follow-up, compared to those in the 1st quartile. Interestingly, subjects with the highest age-corrected LTL had a higher median concentration of vitamin B6 and a lower plasma HCY concentration than all others. In addition, LTL was higher in subjects with plasma HCY concentrations below the cut-off of 12 μmol/L, recommended by the German-Austrian-Swiss ‘DACH-Liga Homocysteine’ [98] than in those above. Furthermore, age-corrected relative LTL was significantly correlated with HCY and vitamin B6.

B12, an essential co-factor for the two enzymes, methionine synthase and methylmalonyl CoA mutase, is also associated with mortality, and its deficiency is common in elderly subjects [99]. Elevated concentrations of HCY and methylmalonic acid (MMA) indicate functional B12 deficiency [100]. Together with folate, B12 has been linked to genome stability and telomere function [101]. Both vitamins are required for the production of methyl groups and nucleotides [98,102]. While nucleotides are the building blocks for DNA (including telomeres), methyl groups are needed for the methylation of DNA, a key epigenetic mechanism that regulates gene expression and histone modifications, which are involved in gene regulation, DNA repair, and chromosome condensation [103]. Aberrant methylation of subtelomeric DNA appears to impact the length, structure, and function of telomeres [104,105,106]. Considering the aforementioned functions of these two vitamins, B12 and folate deficiencies could promote telomere shortening and telomere dysfunction through DNA hypomethylation and impaired nucleotide synthesis. B12 is further required for the maintenance of anti-oxidative defense [107]. Until now, the relationship between LTL and B12 has not been studied comprehensively, and the few existing data are inconsistent [63,96,97]. In the LURIC study [108], B12 was associated with all-cause-mortality, LTL and high sensitive C-reactive protein (hsCRP) in a non-linear fashion [108].

## 7. Markers of Inflammation, Oxidative Stress, HCY, LTL, and Mortality

Chronic smoldering inflammation appears to be the key driver of atherosclerosis and CVD beyond traditional risk factors. In addition, inflammatory processes in blood vessels are, at least partially, promoted by accelerated cellular aging in vascular tissue. This may result in a vicious cycle that promotes atherosclerosis, independently from other cardiovascular risk factors.

The measurement of LTL has been proposed as a valuable tool to estimate the individual inflammatory burden [109]. Long-lasting tissue stress or malfunction can induce the secretion of pro-inflammatory cytokines by macrophages that stimulate the proliferation and differentiation of peripheral lymphocytes [110]. The increased division rate of lymphocytic precursor cells, increased oxidative stress, and other factors promote a reduction of LTL [27,109]. Although this effect is counteracted by telomerase, chronic inflammation can overwhelm the compensatory capacity of telomerase, leading to a net-reduction of LTL [111,112]. Therefore, accelerated cell proliferation in conjunction with chronic inflammation is considered a major reason for rapid telomere attrition in elderly subjects [3]. As mentioned before, inflammation is typically associated with elevated oxidative stress. In dividing cells, oxidative stress increases the telomere loss rate due to the end-replication problem and impaired access of DNA repair complexes to DNA damage sites in the t-loop [113]. The end-replication problem refers to the loss of telomeric DNA with every cell division, which is caused by the incomplete DNA replication at the lagging strand. While synthesis of the new leading strand is a continuous process from the beginning through to the very end of the template, synthesis of the lagging strand is fragmented. For every re-initiation of DNA synthesis at the lagging strand, a short RNA primer is needed. Later, these primers are removed, and the gaps filled with DNA. The gap left by the removal of the most distal primer remains, resulting in a G-rich overhang on one of the chromosomes ends and finally in a shortening of the telomere. It seems obvious that telomeric DNA is lost more rapidly when cells divide more frequently. However, this effect does explain only a fraction of the total telomere shortening effect. The limited access of DNA repair complexes to DNA damage sites in the t-loop has been proposed as another driver of telomere shortening. While DNA damages, such as basic sites and single-strand breaks, are readily repaired all over the genome, the three-dimensional structure of the t-loop hampers this process in the telomeric region. As a result, increased DNA damage in the presence of oxidative stress accelerates the accumulation of DNA damage sites in the t-loop and accelerates the loss of telomeric DNA [113].

An association between short LTL, chronic inflammatory diseases, and oxidative stress has been reported in several studies [64,114,115] and chronic inflammation has been proposed as a functional link between CVD and telomere length [109]. This hypothesis is supported by the observation that patients with rheumatoid arthritis (RA) have an increased risk of CVD [116,117] that goes along with chronic systemic inflammation, elevated oxidative stress and low LTL [65,118]. Furthermore, Sampson et al. reported faster LTL shortening in patients with type 2 diabetes mellitus, which was combined with increased urinary excretion of 8-hydroxy-2′-deoxyguanosine (8-OHdG), a surrogate marker of oxidative DNA damage [119]. In line with this observation, Masi et al. [120] reported a lower LTL in type 1 and 2 diabetics with a reduced total antioxidant capacity in plasma. Patients with short lymphocyte telomeres and a low plasma antioxidant activity were also characterized by a higher risk of incident ischemic heart disease over a follow-up period of 10 years.

Telomere shortening and oxidative stress are important drivers of atherosclerosis. The nicotinamide-adenine dinucleotide phosphate (NADPH) oxidases are major vascular sources of ROS and can be found in endothelial cells, smooth muscle cells, fibroblasts, monocytes and macrophages [121]. Vascular NADPH oxidases, and in particular, the phagocytic NADPH oxidase isoform (Nox2), promote the incidence of atherosclerosis [122,123] and the progression of atherosclerotic lesions [124,125]. Pejenaute et al. [126] reported that TL is inversely correlated with NADPH oxidase-mediated superoxide production, with 8-OHdG levels, and with carotid intima-media thickness (IMT). Asymptomatic patients with plaques had lower TL and higher values of plasma 8-OHdG, and superoxide production than individuals without plaques. The authors conclude that phagocytic NADPH oxidase may be involved in oxidative stress-mediated telomere shortening and that this axis may be critically involved in human atherosclerosis.

Billard et al. [127] investigated the cause of different types of stress, namely mitochondrial metabolic compromise, associated with ROS production, and replicative senescence, activated by extreme telomere shortening. It has been studied how replication stress-induced damage of telomeric DNA (telDNA) and mitochondrial DNA (mtDNA) can be considered as a common origin of senescence with consequences on aging. mtDNA and telDNA share common features that reflect a high degree of replicative stress, such as G-quadruplexes, D-loops, RNA—DNA heteroduplexes, epigenetic marks. Regulated by a feedback loop, specialized telomeric proteins, such as TERT (telomerase reverse transcriptase) and TERC (telomerase RNA component), or TIN2 (telomerase associated protein), shuttle from telomeres to mitochondria, where they modulate mitochondrial metabolism and the production of ROS [127].

ROS are seen as important endogenous sources of telomeric damage. Among the different subcellular compartments, mitochondria are the main source of ROS, with approximately 1–3% of the total molecular oxygen consumption transformed into superoxide anions [128]. Aging in primary cells (independent of expression of telomerase) such as T CD8+ lymphocyte [129] or fibroblasts [130], is associated with a gradual increase in ROS production due to progressive mitochondrial failure and is concomitant to telomere shortening. The neutralization of ROS does not restore mitochondrial function but inhibits telomere shortening, suggesting ROS as a driver of telomere shortening [129]. In diabetics, mitochondrial dysfunction appears to be a major source of oxidative stress that promotes accelerated telomere shortening [131].

The results from an own study [66] suggest that oxidative stress and chronic inflammation are important mediators that link HCY, vitamin B, and LTL with mortality. In the LURIC cohort, interleukine-6 (IL-6) and high-sensitive C-reactive protein (hs-CRP) were significantly lower in subjects with the longest telomeres (4th quartile of LTL) compared to those with the shortest telomeres (1st quartile of LTL). In addition, IL-6 and hs-CRP showed significant inverse correlations with vitamin B6 but positive correlations with HCY. Multiple backward regression analyses identified HCY and IL-6 as strong and independent predictors of LTL. Moreover, IL-6, MTHFR genotype, age-corrected RTL, and vitamin B6 were also independent predictors of HCY.

It is well established that high plasma HCY promotes chronic systemic inflammation through the induction of oxidative stress in many tissue types and subsequent intracellular and extracellular damage [132,133,134]. However, the relationship between HCY and chronic inflammation is not just a one-way road. In vitro experiments have shown that pro-inflammatory cytokines, such as interleukin-1β (IL-1β) and TNF-alpha, alter the cells’ redox state and increase the extracellular HCY concentration in a concentration-dependent fashion [135]. Moreover, systemic inflammation increases vitamin B6 catabolism and cellular uptake, resulting in reduced B6 plasma concentrations [136]. Previous studies have proposed a mechanistic link between oxidative stress, systemic inflammation, and telomere attrition [67,137]. The activation of the complement system and an increased formation of ROS are key components that damage telomeric DNA [138]. In a cross-sectional study from our group, advanced glycation end products (AGEs), a surrogate marker of oxidative stress correlated inversely with LTL (unpublished data). Although oxidative stress-induced telomere shortening is not fully understood, several mechanisms seem to be involved. In dividing cells, oxidative stress can amplify the loss of telomeric DNA caused by the end-replication problem. In contrast, the telomeres of post-mitotic cells can directly be damaged by ROS. In vitro studies have shown that oxidative DNA damage impairs recognition and binding of the shelterin proteins telomeric repeat binding factor 1 (TRF1) and telomeric repeat binding factor 2 (TRF2) to telomeric DNA [139]. Furthermore, the different forms of oxidative DNA damage compromise the protective function of telomeres and trigger systemic inflammation and cellular senescence through a senescence-associated secretory phenotype (SASP) [10]. Furthermore, elevated HCY concentrations are associated with hypomethylation of proteins and DNA and hypomethylation leads to altered gene expression and impairs genomic integrity [140,141]. Methylation of DNA promotor regions modifies gene expression and contributes to disease development [142]. Furthermore, HCY-related hypomethylation affects the methylation status of the telomeric and subtelomeric regions and also influences the gene intron region, which often becomes activated in hypomethylation [140].

## 8. Conclusions

LTL, a surrogate marker of TL in the entire body that decreases with age, is a strong predictor of all-cause mortality, death from vascular causes, and incident vascular events. However, the contribution of short TL to the development and progression of subclinical atherosclerosis is insufficiently understood. Oxidative stress and chronic inflammation appear to be important mediators of telomere shortening that can be affected by multiple factors, including B-vitamins, HCY, and others. Despite promising results from epidemiologic studies, pronounced intra- and interindividual variability hamper a wider application of LTL measurements in clinical practice [22,26,143]. Limited comparability between methods and laboratories further compromises the utility of LTL measurements on an individual basis [144,145,146,147,148].

## Figures and Tables

**Table 1:** Essential studies that investigated the role of telomeres in atherosclerosis and cardiovascular disease.

**Topic**	**Reference**	**Type of Study**	**Participants**	**Follow-up**	**Primary Outcome**
**Telomere length & age**	[23]	Longitudinal population-based	510 males and females mean age at baseline 60 y	10 y	LTL reduction of 45.5 bp/y
	[24]	Longitudinal	334 randomly selected flemish males and females, mean age at baseline 51.9 y	average 7.4 y	Significant LTL reduction, results provided as T/S ratio
	[22]	Longitudinal	271 males and females, Caucasian and African Americans, mean age at baseline 31.9 y	average 12.4 y	LTL reduction of 31 bp/y
	[25]	Longitudinal	75 Dutch men, mean age at baseline 77.6 y	7 y	LTL reduction of 45.5 bp/y
	[26]	Longitudinal	67 children, mean age at baseline 11.4 y, 99 of their parents, mean age at baseline 43.4 y	14 y	LTL reduction in children 40.7 bp/y LTL reduction in adults 20.3 bp/y
	[20]	meta-analysis of 124 cross-sectional studies	124 cross-sectional studies, age range 0–104 y, participants (range): 23–12,409	n/a	weighted mean loss rate: 21.9 bp/y weighted median loss rate: 30.3 bp/y
**Telomere length &** **all-cause mortality**	[21]	prospectice cohort study	3316 patients hospitalized for elective coronary angiography, mean age 62.7 y	median 9.9 y	LTL quartiles 2–4 vs. 1 (shortest telomeres): HR(95% CI) 0.82 (0.71–0.92)
	[15]	prospectice cohort study	8633 females from the Nuses Health study, mean age at baseline 59 y, 3566 males and females from the ESTHER study, mean age at baseline 61.9 y	18.4 y	shortest vs. longest LTL quintile: HR (95% CI) 1.23 (1.04–1.46)
	[17]	prospectice cohort study	64,637 participants from the Copenhagen City Heart Study, Copenhagen General Population Study	median 7 y	shortest vs. the longest decile of LTL: HR (95% CI) 1.40 (1.25–1.57)
	[38]	meta-analysis of 25 prospective cohort studies	12,083 participants, 2517 deaths	n/a	per 1 SD LTL decrement: HR (95% CI) 1.09 (1.06-1.13); shortest vs longest LTL quartile: HR (95% CI) 1.26 (1.15–1.38)
**Telomere length &** **cardiovascular mortality**	[14]	populatin-based prospectice cohort study	800 males and females, mean age at baseline 62.7 y	10 y	shortest vs. the longest tertile of LTL: HR 3.04 (95% CI: 1.13–8.19)
	[21]	prospectice cohort study	3316 patients hospitalized for elective coronary angiography, mean age 62.7 y	median 9.9 y	LTL quartiles 2–4 vs. 1 (shortest telomeres): HR(95% CI) 0.84; (0.72–0.97)
	[15]	prospectice cohort study	8633 females from the Nuses Health study, mean age at baseline 59 y, 3566 males and females from the ESTHER study, mean age at baseline 61.9 y	18.4 y	shortest vs. longest LTL quintile: HR (95% CI) 1.10 (0.88–1.37)
	[17]	prospectice cohort study	64,637 participants from the Copenhagen City Heart Study, Copenhagen General Population Study	median 7 y	per 200 bp reduction of LTL: HR(95% CI) 1.02 (1.01–1.03)
**Telomere length &** **atherosclerosis**	[50]	prospectice cohort study	2819 participants, were free of overt CVD, mean age at baseline 38.5 y	average 5.5 y	shortes vs. longest LTL tertile: HR(95% CI) for incident plaque 1.49 (1.09–2.03) HR(95% CI) plaque progression 1.61 (1.26–2.07)
	[14]	populatin-based prospectice cohort study	800 males and females, mean age at baseline 62.7 y	10 y	shortest vs. the longest tertile of LTL: HR 3.18 (95% CI: 1.66–6.08) composite CVD end points (stroke, myocardial infarction, vascular death)
	[58]	cross-sectional	1459 participants without CVD at recruitment, age at baseline 40–54 y	n/a	Average LTL and short telomere load are no significant predictors of total and femoral plaques
	[57]	cross-sectional	325 subjects free of diabetes, coronary artery disease, stroke and cancer, age 40–64 years	n/a	Shortest vs. longest tertile of LTL: OR (95% CI) 2.36 (1.23–4.52) for having coronary artery calcification (after adjustment for age, race, gender, metabolic syndrome)
	[52]	cross-sectional	2509 participants withoutestablished CVD, aged approximately 35–55	n/a	LTL is neither an independent determinant of intima-media-thickness nor plaque presence
**Telomere length, HCY** **and B-vitamins**	[61]	meta-analysis of 26 studies	n/a	n/a	estimated RR (95% CI) for coronary heart disease events associated with each 5-μmol/L increase in homocysteine 1.18 (1.10–1.26)
	[62]	cross-sectional population-based cohort study	1319 healthy subjects, mean age 49 y	n/a	adjusted LTL difference in the highest and lowest tertile of HCY was 111 base pairs (corresponding to 6.0 years of telomeric aging)
	[63]	cross-sectional cohort study	1715 females	n/a	no sognificant association between LTL, HCY and B-vitamins
**Telomere length,** **oxidative stress** **and inflammation**	[64]	cross-sectional and prospective cohort-study	489 type 2 diabetics, mean age 67 y	10 y	at baseline correltation between LTL and total antioxidant staus (r = 0.106, *p* = 0.024), lower TAOS and shorter LTL at baseline predicted increased risk of incident ischemic heart disease
	[65]	cross-sectional	176 patients with rheumatoid arthritis and 1151 controls	n/a	in rheumatoid arthritis patients significantly lower LTL, LTL unrelated to disease duration, CRP or rheumatoid factor
	[66]	cross-sectional	2968 patients hospitalized for elective coronary angiography, mean age 63.5 y	n/a	Subjects with the longest telomeres had lower concentrations of HCY, IL-6, and hs-CRP
	[67]	cross-sectional	1962 adults; age range: 70–79 y	n/a	OR (95% CI) for LTL in the shortest tertile: 1.3 (1.1–1.7) for subjects with IL-6 in top tertile 1.5 (1.2–1.9) for subjects with TNF-a in top tertile 1.8 (1.3–2.4) for subjects IL-6 + TNF-a in top tertile
